# Surgical Treatment, Rehabilitative Approaches and Functioning Assessment for Patients Affected by Breast Cancer-Related Lymphedema: A Comprehensive Review

**DOI:** 10.3390/medicina61081327

**Published:** 2025-07-23

**Authors:** Paola Ciamarra, Alessandro de Sire, Dicle Aksoyler, Giovanni Paolino, Carmen Cantisani, Francesco Sabbatino, Luigi Schiavo, Renato Cuocolo, Carlo Pietro Campobasso, Luigi Losco

**Affiliations:** 1 Department of Experimental Medicine, University of Campania “Luigi Vanvitelli”, 80138 Naples, Italy; 2 Physical Medicine and Rehabilitation Unit, Department of Medical and Surgical Sciences, University of Catanzaro “Magna Graecia”, 88100 Catanzaro, Italy; 3 Research Center on Musculoskeletal Health, MusculoSkeletalHealth@UMG, University of Catanzaro “Magna Graecia”, 88100 Catanzaro, Italy; 4 Department of Plastic Reconstructive and Aesthetic Surgery, Faculty of Medicine, Istanbul University, 34134 Istanbul, Turkey; 5 Unit of Dermatology, IRCCS Ospedale San Raffaele, Università Vita-Salute San Raffaele, 84131 Milano, Italy; 6 Dermatology Clinic, Department of Clinical Internal, Anesthesiological and Cardiovascular Sciences, University of Rome “Sapienza”, 00161 Rome, Italy; 7 Department of Medicine, Surgery and Dentistry, University of Salerno, 84081 Baronissi, Italy

**Keywords:** breast cancer-related lymphedema, lymphedema, BCRL, plastic surgery, rehabilitation, manual lymphatic drainage, vascularized lymph node transfer, LVA, lymphatic surgery, ICF, disability assessment, quality of life

## Abstract

*Introduction:* Breast cancer therapy is a common cause of lymphedema. The accumulation of protein-rich fluid in the affected extremity leads to a progressive path—swelling, inflammation, and fibrosis—namely, irreversible changes. *Methods:* A scientific literature analysis was performed on PubMed/Medline, Scopus, Web of Science (WoS), the Cochrane Central Register of Controlled Trials (CENTRAL), and the Physiotherapy Evidence Database (PEDro) from inception until 30 June 2024. *Results:* Breast cancer-related lymphedema (BCRL) is indeed an important healthcare burden both due to the significant patient-related outcomes and the overall social impact of this condition. Even though lymphedema is not life-threatening, the literature underlined harmful consequences in terms of pain, infections, distress, and functional impairment with a subsequent and relevant decrease in quality of life. Currently, since there is no cure, the therapeutic approach to BCRL aims to slow disease progression and prevent related complications. A comprehensive overview of postmastectomy lymphedema is offered. First, the pathophysiology and risk factors associated with BCRL were detailed; then, diagnosis modalities were depicted highlighting the importance of early detection. According to non-negligible changes in patients’ everyday lives, novel criteria for patients’ functioning assessment are reported. Regarding the treatment modalities, a wide array of conservative and surgical methods both physiologic and ablative were analyzed with their own outcomes and downsides. *Conclusions:* Combined strategies and multidisciplinary protocols for BCRL, including specialized management by reconstructive surgeons and physiatrists, along with healthy lifestyle programs and personalized nutritional counseling, should be compulsory to address patients’ demands and optimize the treatment of this harmful and non-curable condition. The Lymphedema-specific ICF Core Sets should be included more often in the overall outcome evaluation with the aim of obtaining a comprehensive appraisal of the treatment strategies that take into account the patient’s subjective score.

## 1. Introduction

Breast cancer (BC) is the most common type of cancer and it is commonly diagnosed in women; it poses a significant health burden globally, with over 2 million new cases diagnosed in 2022 [1]. There are several therapeutic options for breast cancer patients. Surgical treatment, namely total or partial mastectomy and sentinel node biopsy or axillary lymph node dissection, could be combined with radiotherapy and/or chemotherapy and/or hormone therapy depending on the stage of the disease, the breast cancer subtype, and the clinicopathological characteristics of the patients. Unfortunately, breast cancer treatment can be associated with noteworthy complications such as hematoma, infection, seroma, cellulitis, and most specifically, lymphedema, due to the disruption of the axillary lymphatic vessels leading to the accumulation of protein-rich fluid in the interstitial space [2,3].

To date, the increased prevalence of breast cancer-related lymphedema is due to improved screening and therapeutic strategies for breast cancer, along with the subsequent higher survival rates [4,5,6,7]. Patients with BCRL often report upper-limb weighting, a decreased range of motion, pitting edema, pain, recurrent episodes of cellulitis; in later stages, elephantiasis and cutaneous angiosarcoma could occur. Moreover, patients can go through psychosocial stress related to anxiety, depression, and body image-related disturbance that leads to a dramatically affected quality of life (QoL) [8,9,10,11].

Given its chronic nature, lymphedema leads to financial burdens for patients, caregivers, and the society as a whole. In a comparison between breast cancer patients without lymphedema and BCRL patients, a 7-fold greater average healthcare charge per patient ($141,388 vs. $21,141 per patient, respectively) was demonstrated in a study that lasted two years after cancer removal; moreover, women with complicated lymphedema experienced 5-fold greater all-cause and 2-fold greater non-lymphedema hospital admissions compared with women without complicated lymphedema. This high rate of hospitalization resulted in substantially higher healthcare charges [12]. Evidence suggests that surveillance and early identification methods are more economically advantageous compared to lymphedema to manifest [13]. Even though not inherently life-threatening, these changes significantly impact quality of life (QoL), a health indicator whose importance is often underestimated. Implementing QoL and functionality assessment could be crucial to defining therapeutic strategies by detecting environmental factors such as facilitators of or barriers to functioning and action performance [14,15,16].

Currently, since there is no cure, and the therapeutic approach to BCRL is aimed at slowing the disease’s evolution and preventing derivative complications, in this context a prompt diagnosis is pivotal to settling on an efficacious protocol through physical therapy or surgery [17,18]. The aim of well-timed intervention for BCRL is to reduce swelling, firmness, and limb circumference, and to mitigate joint and muscle ache, reduce cellulitis episodes, lower therapeutic plan costs, motivate patients to continue therapy, and increase QoL [19,20,21,22].

In this scenario, international guidelines have outlined a comprehensive conservative approach commonly defined as Complex Decongestive Therapy (CDT). It includes manual lymph drainage (MLD), compression garments, specialized exercises, skin care, and self-education [23]. CDT is widely accepted as the universal first-line therapy for lymphedema of the limbs, and surgical therapy is usually decided upon if or when the conservative approach is no longer successful.

On the one hand, there is a chance to eliminate the affected skin and subcutaneous tissue that show fibrotic changes through debulking techniques. In the early stages, when the fibrotic changes are not yet evident, suction-assisted lipectomy (SAL), is exploited for adipose tissue collection; however, when fibrosis is noticeable, a radical resection is needed. On the other hand, recent progress in microsurgical techniques parallel to a thorough comprehension of lymphedema’s physiopathology ha established new physiological procedures that aim at reestablishing the lymphatic outflow, namely Lymphaticovenular Anastomosis (LVA) and vascularized lymph node transfer (VLNT) [24,25].

Nowadays, a lack of consensus among the experts regarding the most effective protocol for the treatment of BCRL should be noted: the intrinsic peculiarities of lymphedema itself with its manifold pathophysiology and presentations and the heterogeneity in lymphedema staging are serious drawbacks in terms of the pathway toward setting up an evidence-based treatment algorithm.

We hereby provide a comprehensive overview of the current strategies for treating BCRL from the standpoint of plastic reconstructive surgeons and physical and rehabilitative medicine, focusing on recent advancements in evidence-based interventions and functioningl assessment.

## 2. Research Methodology

A scientific literature analysis was performed on PubMed/Medline, Scopus, Web of Science (WoS), the Cochrane Central Register of Controlled Trials (CENTRAL), and the Physiotherapy Evidence Database (PEDro) from inception until 30 June 2024 using the following Mesh terms: “Breast Cancer Related Lymphedema”, “Lymphedema”, “Breast Cancer”, “Physical Therapy”, “Rehabilitation”, “Manual Therapy”, “Manual lymphatic drainage”, “Exercise”, “Functioning”, “Anastomosis”, “Lymphaticovenular Anastomosis”, “Vascularized Lymph Node Transfer”, “Liposuction”, and “Lymphatic surgery”. The literature search was performed between October 2023 and July 2024 by two independent reviewers who, thereafter, independently examined the studies for eligibility. If agreement was not reached, a third reviewer was solicited. The inclusion criteria were the following: full-text studies in English assessing adult patients (aged > 18 years) with BCRL or at high risk of lymphedema. The exclusion criteria were studies in languages other than English, studies without the full text available, studies involving animals, conference abstracts, and master’s or doctoral theses. A qualitative method was used for data extraction and data synthesis. Both data extraction and synthesis were separately carried out by two reviewers. In case of disagreement, synthesis was independently performed by two reviewers. If consensus was not reached by discussion, a third reviewer was appealed to.

## 3. Lymphedema: Etiology, Pathophysiology, and Hallmarks

Lymphedema is a chronic tissue swelling caused by the accumulation of protein-rich lymphatic fluid in the interstitial space, which is usually drained via the body’s lymphatic system. Lymph node and/or lymphatic vessel impairment causes an imbalance between the rate of interstitial fluid formation and the lymphatic transport capacity [26,27].

Axillary lymph node dissection (ALND), an increased number of dissected nodes, right-sided and hypofractionated radiotherapy with regional node irradiation (RNI), and obesity BMI ≥ 30 kg/m^2^) were found to be significant risk factors [28]. A genetic polymorphism has also been detected in patients with BCRL, suggesting a genetic predisposition to lymphedema [29,30]. Immediate or delayed breast reconstruction by autologous flap or prosthesis may reduce the risk of BCRL [31]. In secondary studies, the incidence of BCRL has been shown to be four times lower in patients receiving sentinel LN biopsy (5.6%) compared to ALND (19.9%) [4]. Likewise, Kilbreath et al. [5] reported that the incidence of BCRL for patients who have had less than five nodes removed was at 3.3%, while for patients with five or more nodes removed, the incidence was 18.2%. Moreover, the sentinel lymph node biopsy (SLNB)+ regional nodal irradiation (RNI) group had a significantly lower BCRL risk than the ALND + RNI group [28].

Locoregional adjuvant radiation therapy has also been shown to be a significant factor associated with lymphedema; however, conflicting outcomes have been reported. On the one hand, Shaitelman et al. [6], in a systematic review, showed an increased risk of BCRL with the addition of RNI after ALND compared to ALND alone. On the other hand, a recent study on 1369 women showed no significant differences between ALND vs. ALND + RNI or SLNB vs. SLNB + RNI [28].

Therefore, surgical training plays a crucial role in reducing the incidence and severity of BCRL by improving surgical techniques. Training in minimally invasive approaches, namely SLNB and Axillary Reverse Mapping (ARM) could lead the surgeon to remove fewer lymph nodes, thus reducing the lymphatic drainage disruption; moreover, enhanced surgical skills in axillary surgery could minimize the damage to lymphatic tissue [32,33].

Lymphedema is defined as “primary” if not connected to any medical condition. It could be congenital or arise in adulthood due to congenital abnormalities or developmental defects in the lymphatic system [28]. When lymphatic impairment is caused by direct trauma, infection, chronic venous insufficiency, radiotherapy, or, as is most common, after surgical intervention for cancer, the lymphedema is defined as “secondary”. Globally, according to World Health Organization (WHO) estimates, secondary lymphedema affects 170 million people [30,31,34].

**Figure 1:**
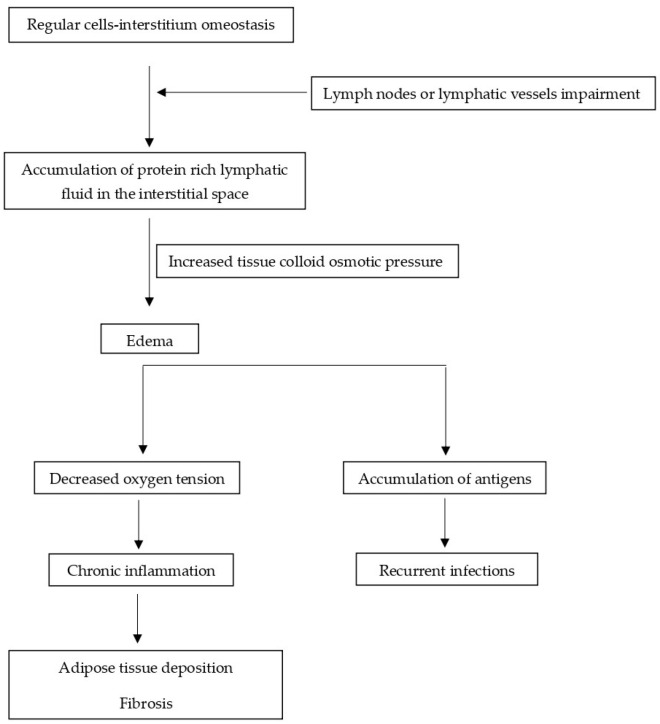
Pathophysiology of Lymphedema.

BCRL is a form of secondary upper-limb lymphedema that is caused by breast cancer treatment. Soon after breast cancer surgery, if the edema spreads into the interstitium of the involved limb, the patient could complain of a feeling of heaviness. Then, the edema gradually increases and becomes evident; an evolution from pitting to non-pitting edema is reported and, later, transitioning to adipose tissue proliferation; then, when fibrosis takes place, the lymphatic system activity becomes even more impaired (Figure 1).

## 4. Preoperative Clinical Assessment and Imaging

An accurate diagnosis of BCRL requires a complete clinical history (including risk evaluation and medications that could cause edema), a physical examination, and physiologic measures [35] avoiding subjective swelling interpretations [36].

Lymphedema is clinically evident when tissue swelling is visible or palpable, while subclinical lymphedema is diagnosed as an increase in volume of at least 3%, measuring the perimeter of the affected upper limb in comparison to the volume of the contralateral limb using bioimpedance spectroscopy (BIS) [37,38]. At physical examination the size, presence of scars, skin condition, and sensation of the affected limb should be evaluated. Affected patients may display skin blush, hyperkeratosis, and stiffness of soft tissues [39]. Subjective symptoms are not experienced by all patients; such evidence includes heaviness, torpor, swelling, firmness, and limb weakness. In the early stages of lymphedema, a temporary skin depression (known as “pitting edema”) appears after exerting pressure with the fingertip. On the other hand, non-pitting edema could be present in later stages: adipose tissue appears augmented with various degrees of fibrosis [39].

The preoperative assessment of lymphedema requires a measurement of the limb volume. To date, a universally accepted method is missing. Most commonly, a tape is used for limb circumferential measurements in order to apply the truncated cone formula. Other techniques include water displacement, perometry, and 3D laser scan [40,41,42].

In the case of subclinical BCRL, bio-impedance spectroscopy (BIS) is effective. A significant multi-center randomized controlled trial performed by Ridner et al. [43] (the PREVENT study) evaluated the effectiveness of BIS versus tape measurement (TM) in the early detection and treatment of BCRL. According to this study, BIS screening in the early stages can prevent lymphedema.

It is suggested that all patients, even those who do not report subjective symptoms, should undergo screening for lymphedema [30] for 2–6 years after the treatment [44]. Therefore, it was found that continuous monitoring and early diagnostics are cheaper if compared to waiting for signs or symptoms to display [21]. Commonly, BCRL is defined as an increase of 2 cm or more in the arm circumference [45] and can develop from 2 to 10 years after mastectomy and axillary lymphadenectomy [4,19]. With such premises, a diagnosis in the subclinical phase could be crucial for prompt lymphedema management.

Along with limb measurement techniques and devices, imaging plays a fundamental role in the preoperative assessment. Lymphoscintigraphy provides information about lymphatic anatomy and function and is considered the gold standard for lymphedema diagnosis [40,41]. Indocyanine green (ICG) lymphography makes it possible to examine the function of the superficial peripheral lymphatic system prior to surgery, identifying reliable lymphatic vessels for LVA or the recipient site of VLNT [46].

Among imaging techniques, the availability of ultrasound (US) as an easily accessible, non-invasive technique, which does not expose patients to ionizing radiation, is also worth noting. In the setting of lymphedema, US can provide high-resolution assessment of all tissue layers involved in the pathophysiological process, from the epidermis to subcutaneous layers [47]. Specifically, signs that can be assessed include skin thickness and edema, and they are also quantifiable in terms of excess volume compared to the opposite limb. Furthermore, more advanced quantitative modalities, such as sonoelastography, may offer additional means of investigating BRCL and its progression [48].

Magnetic resonance lymphangiography (MRL) offers information about superficial and deep lymphatic vessels; moreover, adjacent structures are also studied. Therefore, its use is relevant: it enhances pre-surgical preparation, reduces surgical time, and allows for smaller incisions [46,49].

## 5. Staging

Secondary lymphedema, including BCRL, can be classified into several stages, according to the clinical symptoms, pathological changes, and imaging [50,51,52,53]. The classification provided by the International Society of Lymphology (ISL) is the most adopted and identifies three clinical stages of lymphedema (Table 1). At stage 0 an impaired lymphatic transport swelling is not evident, but some subjective symptoms might be present. It is recorded as subclinical lymphedema and edema could arise even after many years. At stage I early swelling occurs, but it subsides with limb elevation; also pitting may occur. Stage II is characterized by significant protein-rich fluid accumulation and adipose tissue deposition; swelling rarely subsides with limb elevation and pitting is evident. The amount of subcutaneous adipose deposition increases in late-stage II until fibrotic transformation; pitting may not occur. Stage III is also defined as “lymphostatic elephantiasis”: extensive fat deposition and fibrosis occur; pitting is absent. Skin thickening, acanthosis, and a warty overgrowth often develop. Several stages of lymphedema could be evident at the same time, reflecting different lymphatic areas in a single arm. The stage of the disease will address treatment options.

## 6. Functioning and Quality of Life Assessment in Patients Suffering from Lymphedema

Objective assessment of the limb volume can be performed with several valid and reliable methods. However, determining only the volume does not allow for an accurate estimation of the real burden of this chronic health condition, which significantly affects mobility, personal care, occupation, domestic life, and social interactions. Indeed, function impairments, activity limitation, and participation restrictions can affect the QoL of breast cancer patients. Moreover, patients with a chronic condition are usually at risk of depression and job loss [12,54,55,56,57].

Including quality of life (QoL) measures in clinical practice could be useful for assessing the impact of chronic edema on the individual, demonstrating treatment-related changes, and guiding treatment decisions. Several lymphedema-specific QoL measures have been reported: ULL-27 for upper-limb lymphoedema [58], FLQA-L for lymphoedema of the arms and legs [59], the Wesley Clinic Lymphoedema Scale (WCLS) or post-mastectomy lymphoedema [60], and a QoL measure for limb lymphedema (LYMQOL).

LYMQOL is commonly used in clinical practice: it is a patient-completed questionnaire developed in the UK and includes two separate tools for arm and leg lymphedema. The questions cover four domains: symptoms, body image/appearance, function, and mood. However, according to the concept of the “disability paradox” [61], poor functioning does not necessarily associate with QoL, commonly defined as good health, well-being, and life satisfaction. It can represent a bias in the attitude and expectations of people and healthcare workers towards people with chronic health conditions, whose personal experience and perspective are underestimated. Therefore, not only QoL, but also functioning, should be assessed to guarantee a global evaluation of lymphedema patients.

An international instrument to assess functioning and disability is the International Classification of Functioning, Disability and Health (ICF), provided by the WHO; it is based on the evaluation of four domains: body functions, body structures, activity and participations, and environmental factors. To facilitate the application of the ICF in rehabilitation medicine, lymphedema-specific ICF Core Sets—a selection of relevant ICF categories—were developed in 2015 [62]. There are two Core Sets for lymphedema (brief and comprehensive), each divided into three regions including the upper extremity, the lower extremity, and the midline. The Brief Sets can be used during individual treatment, and the Comprehensive Sets in multidisciplinary settings. ICF Core Sets can be self-administered and scored by the patient; responses are indicated on a three-point rating scale (satisfied, neutral, dissatisfied). All four domains of the ICF are explored by the Core Sets for lymphedema. However, activities and participations and body functions alone make up almost the whole checklist since the disability caused by lymphedema does not so much affect the “mobility” or body structures, but rather the activity and participation in daily life. Therefore, the complete application of the Core Sets requires an exhaustive medical history collection focused on both the description of the daily environment and personal feelings.

Among the others, the disability assessment schedules used for lymphedema, the Lymphedema Functioning, Disability and Health questionnaire for the upper limb (Lymph-ICF-UL) was first developed in 2011 and revised in 2019 to assess problems in functioning including the impairments, activity limitations, and participation restrictions of patients with BCRL [63]. De Vrieze et al. [63] found that the revised Lymph-ICF-UL questionnaire was appropriate and useful in clinical practice as it displays clinical properties from very good (reliability) to good (validity). Thereafter, the questionnaire has been used to monitor the long-term effects of therapy and self-care from a biopsychosocial health perspective, taking into account the patient’s point of view and functioning in addition to the clinical evolution of the disease.

## 7. Management Strategies for Breast Cancer-Related Lymphedema

The prompt detection and diagnosis of lymphedema is a determinant in lymphedema management, allowing for early specific treatments. The increase in limb volume should be identified quickly with the aim of treating lymphedema in its earlier stages, avoiding drawbacks and progressive disease evolution [64]. Management protocols include either conservative or invasive surgical treatments.

### 7.1. Conservative Therapy

#### 7.1.1. Complete Decongestive Therapy

Conservative treatment is also known as non-operative treatment. Complex Decongestive Therapy or Complete Decongestive Therapy (CDT) is a two-stage treatment program and, according to the latest ISL Consensus, is considered the standard of care for lymphedema. [51]. Phase 1 consists of skin care and specific light manual massage (manual lymphatic drainage—MLD). Deeper techniques, such as muscle pumping exercises and compression with multilayered bandage wrapping, are required for patients classified above Stage I. Phase 2 starts soon after Phase 1 and is aimed at preserving and optimizing the results obtained in the first phase. Compression by a low-stretch elastic stocking or sleeve is considered the cornerstone of CDT, but Phase 2 also includes skin care, continued exercise, and MLD as needed. Different methods, times, and durations of this treatment can be provided [65].

Tantawy et al. [66] matched the outcomes of compression wear and Kinesio taping in patients with BCRL. In this RCT, the analysis of the two groups showed significant differences in terms of lymphedema volume, upper-limb function, muscle strength, and quality of life. Moreover, the improvement in upper-limb function, muscle strength, and quality of life was significant (*p* < 0.05) only for the Kinesio tape group. On the other hand, a recent systematic review [67] demonstrated that Kinesio taping did not significantly reduce the upper-limb volume in BCRL women, although it could increase the flow rate during the passive exercise. Indeed, further high-quality studies are necessary in order to definitely include this rehabilitative technique in the management of BCRL subjects.

Torres-Lacomba et al. [68] conducted a randomized, single-blind, clinical trial comparing the amount of lymphedema volume reduction in BCRL patients treated with four types of bandages versus patients treated with Kinesio tape. After three weeks of treatment, significant differences were found in terms of lymphedema volume. The group undergoing compression with two layers combining inelastic and elastic bandaging experienced a larger improvement in volume reduction (59.5%) but more discomfort, while Kinesio tape was the most comfortable compression strategy (*p* < 0.001). Similarly, de Sire et al. evaluated the reduction in lymphedema volume after treatment with an instantly adjustable inelastic compression device. They found a significant improvement without relevant drawbacks; therefore, the possible involvement of the instantly adjustable inelastic compression device was suggested [23,69]. Ayhan et al. [70] investigated the safety of multilayer bandaging in CDT, evaluating if it may increase tissue pressure resulting in nerve entrapments. CDT was found to be effective and safe according to volumetric calculations, US measurements of tissue thicknesses, and median nerve size. Moreover, Gokçe et al. [71] used the Finger Tapping Task to assess fine motor performance in BCRL patients: They reported that CDT is effective in improving upper-extremity fine motor function. Ezzo et al. [72] reported that MLD is safe and may offer additional benefit to compression bandaging for swelling reduction in patients with mild-to-moderate BCRL, compared to individuals with moderate-to-severe BCRL.

However, a recent review of systematic reviews pointed out a great variability in CDT delivery and outcomes. CDT’s efficacy in BCRL is supported by current evidence, but MLD and exercise provide limited support for volume reduction, as sustained by moderate- to low-quality systematic reviews. On these bases, the need to standardize procedures, staging criteria, and outcome measures in research is clear [20,73], and future research should focus on improving evidence-based BCRL management.

#### 7.1.2. Physical Agent Modalities

Physical agent modalities have recently been proposed in the management of BCRL women to achieve lymphedema volume reduction and chronic inflammation regulation. Interesting but not always significant results have recently been provided.

In particular, Lee et al. [74] evaluated the effects of combined CDT and extracorporeal shockwave therapy (ESWT) (two times a week for 3 weeks) in the management of fibrotic lesions. A significant recovery in lymphedema was observed. Moreover, the authors reported significant differences compared to the control group (CDT only) in terms of limb volume, the extracellular water-to-total body water ratio, and skin thickness. Low-level laser therapy has also been proposed as a promising tool for BCRL patients due to its anti-inflammatory and lymph-angiogenetic effects [75]. However, Kilmartin et al. [76] recently evaluated the outcomes of CDT combined with low-level laser therapy or with sham low-level laser therapy: except for self-reported outcomes, significant results in limb volume reduction were not found.

Pneumatic compression therapy (PCT) involves the application of sequential or intermittent pneumatic compression garments to the affected area. It exerts external pressure and stimulates lymphatic drainage [77], mimicking the natural muscle pumping action, thus promoting the lymphatic flow and reducing swelling. The effectiveness of PCT in lymphedema management has been demonstrated in several studies. Uzkeser et al. [78] performed a systematic review and meta-analysis to evaluate the efficacy of PCT in the treatment of lymphedema. PCT proved effective in reducing limb volume and alleviating symptoms in BCRL patients. However, a consensus about the optimal pressure prescription is missing. In particular, Mosti and Cavezzi reported that low pressure (40–60 mmHg) might offer better results rather than higher compression [79].

#### 7.1.3. Physical Activity and Physical Exercise

Physical activity is defined by the WHO as any movement that uses skeletal muscles and requires energy expenditure. Physical activity intensity can be measured using the Metabolic Equivalent of Tasks (METs); that is, the ratio between the working metabolic rate and the resting metabolic rate. One MET is defined as the energy expenditure when an individual is resting, sitting in a chair in the absence of any other activity [80]. One MET is approximately 3.5 milliliters of oxygen consumed per kilogram (kg) of body weight per minute.

In this context, physical activity is strongly recommended for cancer patients, both for symptom management and for its positive impact on overall survival. [81,82]. For a long time, physical activity was restricted after breast cancer surgery due to concerns that it might increase the risk of upper-limb lymphedema. However, at present, physical activity at any intensity is no longer restricted, since it reduces the risk of lymphedema and has a positive impact on lymphedema volume [83,84]. In the early phases of activity, supervision by a trained professional and progressiveness (number, intensity, and duration) are required.

Physical activity globally improves quality of life with positive effects for physical performance, physical function, reduction in anxiety, depressive syndromes, fatigue, and sleep disturbance; it could improve self-confidence and reduce the risk of breast cancer recurrence and death rate [85,86]. However, there is no consensus on the optimal exercise intervention regimen for patients with breast cancer-related lymphedema (BCRL) [87]. Indeed, the exercise prescription should be evidence-based, but also tailored to the patients’ characteristics.

The recommended amount of activity includes moderate-intensity aerobic exercises (conversation possible, breathlessness). It is outlined as nine MET/h per week (4–5 times, 30 min of brisk walking per week) in combination with muscle strengthening twice a week [88].

On the other hand, it is clear that physical exercise is not the same as physical activity. Specifically, it could be considered a subcategory of physical activity that requires moderate or high energy expenditure, and provides planned and structured sets of repetitive movements aimed at improving or maintaining specific physical levels of functioning. Nevertheless, there is evidence of significant differences between different kinds of exercise. Notably, endurance training promotes oxidative metabolic adaptations, while resistance training allows for an anabolic response, providing muscle hypertrophy and an increase in muscle strength. Moreover, exercise induces autophagy and mitophagy, promoting the removal of damaged mitochondria, improving muscle energy metabolism, and counteracting the mechanisms that lead to the reduction in strength and muscle mass [89,90,91,92,93,94,95,96].

### 7.2. Surgical Therapy

**Figure 2:**
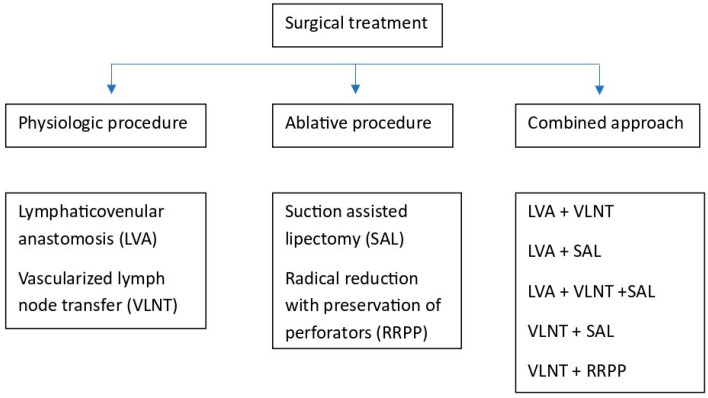
Surgical treatment of BCRL.

The surgical management of lymphedema involves two main techniques: physiologic and reductive approaches. The physiologic procedures, also known as reconstructive, are focused on restoring the physiologic lymphatic anatomy. The reductive procedures aim to remove lymphedematous tissue and fat deposits through liposuction or skin excision [97] (Figure 2 and Table 2).

#### 7.2.1. Physiologic Approach

##### Lymphaticovenular Anastomosis (LVA)

Lymphaticovenular Anastomosis (LVA) or lymphovenous bypass aims to restore local lymphatic circulation by creating one or more anastomoses between the burdened subcutaneous lymphatic vessels proximal to the site of lymphatic block and nearby low-pressure venules. Imaging assessment is usually relevant to managing this procedure (e.g., indocyanine lymphography) [98].

The superiority of LVA versus compressive therapy has been demonstrated by several studies: circumference reduction was found to be 4.1 cm in patients managed with LVA against a 0.8 cm circumference reduction in patients who received compression therapy (*p* < 0.05) [99,100].

However, compression therapy should always be considered as a valuable adjunct in the treatment of upper-limb lymphedema. In fact, it has been reported that postoperative compression immediately following LVA can enhance lymphatic flow without causing damage to the anastomoses [101].

Previously the literature maintained that LVA efficacy is restricted to the early-stage lymphedema due to the availability of functional lymph vessels [101,102]; however, the effectiveness of LVA was recently related also to the later stages. Indeed, it has been reported that preoperative planning for lymphatic surgery should not rely solely on the International Society of Lymphedema staging, as patients with advanced-stage lymphedema who exhibit a high lymphatic fluid transit velocity—assessed using indocyanine green (ICG) lymphography—may still benefit from LVA alone [102].

Patients with BCRL undergoing physiologic procedures such as LVA showed conflicting results regarding objective parameters (e.g., limb CRR or limb volume reduction), but several studies have reported an improvement in patient QoL [103,104]. Cornelissen et al. [105] prospectively assessed improvements in QoL after LVA in women with BCRL. Even though a significant reduction in the mean relative volume was not found, 85% of the patients discontinued compressive stockings. Moreover, after 1 year of follow-up, a statistically significant enhancement in QoL was evident using a validated questionnaire [lymphedema international classification of functioning (Lymph-ICF) questionnaire, Dutch version].

Some notable advantages of LVA include the limited morbidity of the procedure, as the incisions are 2 cm long or even less, and early discharge either on the same day as the surgery or after a 1-day hospitalization. The procedure may be performed under local anesthesia. On the other hand, one limitation of this procedure is the technical challenge of handling vessels and performing anastomoses, as their diameter can be as small as 0.2 mm. Additionally, specialized supermicrosurgical instruments are required [40,106].

##### Vascularized Lymph Node Transfer (VLNT)

VLNT involves the transplantation of functional lymph nodes to restore the natural flow of lymphatic fluid. Unlike LVA, which requires functioning lymphatic vessels, VLNT can be performed even when lymphatic channels are blocked. Therefore, the presence of substantial segmental dermal backflow with minimal or no functional lymphatic vessels seen on imaging indicates the need for vascularized lymph node transplantation. This distribution may also help determine whether to place the lymph node flap in a proximal anatomical (orthotopic) or distal non-anatomical (heterotopic) location [93,107].

It seems that after restoring perfusion and physiological flow, lymph node flaps release growth factors, such as vascular endothelial growth factor (VEGF), which encourage lymphangiogenesis and facilitate the movement of fluid from distal non-functional lymphatic channels into healthy proximal lymphatic vessels. VLNT, functioning as a “pump”, may also aid in lymphatic fluid outlet by rerouting it into the vascular system [108,109,110]. Additionally, the lymph node flap provides benefits by functioning as part of the immune system. After an antigen is presented to the lymph nodes, an immune response could start, thus reducing the risk of infection in the affected limb. It has been reported that most patients who experience relapsing episodes of cellulitis see a substantial decrease in these episodes following VLNT [111].

Node donor sites are axillary, inguinal, submental, supra-clavicular, gastroepiploic, and the recipient site is the axillary, wrist, or elbow. The choice of recipient site is based on the severity of the lymphedema, the availability of recipient vessels, and the surgeon’s preference [112].

The effectiveness of vascularized lymph node transfer for the treatment of lymphedema has been variously demonstrated. Becker et al. [108] assessed 1500 patients with lymphedema stage I to III who underwent VLNT over a minimum follow-up period of 3 years. The results showed a 98% rate of subjective improvement. Forty percent of patients with stage I and stage II lymphedema experienced significant improvement and were able to discontinue conservative treatment. Among patients with stage III lymphedema, 95% showed some improvement, and 98% remained free of infection. However, conservative therapy could not be discontinued in these latter patients.

In a retrospective study by Becker et al. [113], 24 patients with BCRL underwent groin VLNT and were followed for an average of 8.3 years. Patients with lymphedema classified as ISL stage III were excluded from the study. The results showed that 12 patients (50%) experienced a downstaging, while 10 patients (41.7%) had complete resolution of symptoms. However, two patients (8.3%) showed no improvement.

Patel et al. [114] presented a prospective series involving 15 patients treated with a groin VLNT flap and a submental VLNT for upper-extremity lymphedema stages II–IV (a modified lymphedema grading system was employed). A significant reduction in limb volume difference was reported 12 months after surgery, along with a decreased incidence of cellulitis episodes during the observation period. Additionally, patients demonstrated a significant improvement in overall quality of life (QoL), as measured by the lymphoedema quality-of-life tool (LYMQOL) with notable improvements in all specific subdomains, including function, appearance, symptoms, and mood.

Conflicting outcomes regarding the effectiveness of VLNT have also been reported. Gratzon et al. [115] conducted a prospective study on groin VLNT in patients with ISL stage I–II BCRL After a 12-month follow-up, the median percentage reduction in limb volume was 42.73%; however, this difference did not achieve statistical significance (*p* = 0.052).

A recent systematic review and meta-analysis showed that 90% of patients reported a 40% reduction in upper-limb lymphedema volume, along with a decrease in skin infections and an improvement in QoL [116]. Another meta-analysis compared the outcomes of VLNT and LVA in limb lymphedema: although both approaches were effective in the short term, VLNT generated significantly better long-term improvements and a higher probability of discontinuing the use of compression dressings [117].

In comparison with conservative treatments, VLNT demonstrated a relevant therapeutic impact in BCRL patients. Dionyssiou et al. [118] evaluated the effects of groin VLNT combined with physiotherapy in patients with ISL stage II BCRL, compared to a control group that underwent a six-month physiotherapy regimen without surgical intervention. After an 18-month follow-up, 72% of the patients in the VLNT group showed functional lymph nodes on postoperative lymphoscintigraphy. The average differences in volume, infection rates, pain scores, heaviness scores, and overall function scores in patients who received VLNT along with 6 months of physiotherapy were significantly better than those in the group that only had physiotherapy (*p* < 0.001 for all comparisons).

A significant drawback of VLNT is the risk of donor site iatrogenic lymphedema. One approach developed to mitigate this issue is reverse mapping; it helps identify and protect the lymph nodes that primarily drain the affected extremity, ensuring these nodes are preserved during the harvest of groin or lateral thoracic lymph nodes. A radiotracer is injected into the subdermal plane at the web spaces of the affected limb, and ICG is administered into the subdermal plane of the groin or axilla on the same side. During surgery, a gamma probe and ICG dye are used to identify the lymph nodes that mainly drain the donor limb; any lymph nodes showing radiotracer uptake are preserved and not included in the flap harvest. Dayan et al. have reported promising results using this technique [119]. Most notably, to date, the harvest of lymph nodes from the gastroepiploic region has never been linked to iatrogenic lymphedema. The requirement of an abdominal surgeon to perform the harvest is the main shortcoming of such a procedure [120,121].

Across all studies, reconstructive surgical techniques are not directly comparable, as evaluations vary depending on the surgical methods and study designs, and results are occasionally inconsistent. Further research with more rigorous and standardized methodologies is needed to provide clearer insights.

#### 7.2.2. Reductive Approach

The gradual interstitial accumulation of lymph triggers a series of harmful events leading to fat deposition and fibrosis. Once fibrosis and lipodystrophy have developed in the affected limb, managing fluid accumulation alone is no longer effective in reducing limb size. Excisional procedures (also known as ablative or reductive procedures) involve the removal of excess subcutaneous and/or cutaneous tissue along with fluid overload.

In advanced lymphedema, a reductive approach aimed at removing the accumulated fibrofatty tissue can be effective. The soft tissues, located above the level of the deep fascia, are typically edematous and/or fibrotic, and could be surgically removed through direct excision or liposuction; however, lymphatic flow is not restored. In contrast, the physiologic procedure as a single treatment could show a narrow therapeutic effect in patients with advanced-stage BCRL [99].

Ablative procedures like the Charles’ or the Sistrunk procedure achieve the complete removal of fibrotic and subcutaneous tissue through direct excision [122,123]. Given the morbidity associated with these procedures, suction-assisted lipectomy (SAL) and radical reduction with preservation of perforators (RRPP) have become increasingly popular for the treatment of postmastectomy lymphedema [99,120].

##### Suction-Assisted Liposuction (SAL)

Fat accumulation is a crucial finding in lymphedema. Despite intense research, the underlying mechanism behind adipose tissue buildup in lymphedema is still debated. Suction-assisted lipectomy is a widely used technique for removing excess fat from the affected limb. This method is indicated for both upper- and lower-limb lymphedema without pitting edema. It is considered the least invasive of the reductive procedures, and following liposuction, lymphedematous limb volume often becomes comparable to the unaffected side.

Significant reductions in arm size and decreases in cellulitis episodes have been reported after SAL [124,125]. Additionally, SAL has been shown in other studies to achieve a mean excess volume reduction of 109% in the upper extremity (*p* < 0.001) and has proven highly effective in reducing the annual frequency of cellulitis episodes by 87% (*p* < 0.001) [126].

In a recent study, power-assisted liposuction was compared with a combination of compression garments and physical therapy in women with fat-dominant BCRL. The ablative procedure showed more favorable outcomes in terms of quality-adjusted life years (QALYs) than conservative procedures. The relative cost reduction associated with SAL was estimated at $74,487 [127].

On the other hand, since liposuction does not enhance the function of the lymphatic pathway; patients still need to wear compressive clothes lifelong to prevent any recurrence.

##### Radical Reduction with Preservation of Perforators (RRPP)

Radical reduction with preservation of perforators (RRPP) is a modified version of traditional excisional techniques that assumes the perforator flap concept. Cutaneous flaps are raised, and, while the relevant perforators are spared, the subcutaneous tissue is removed. This single-stage procedure provides long-lasting volume reduction and satisfactory cosmetic results. Salgado et al. [128] reported the outcomes of eleven patients with ISL stage IIB BCRL who underwent RRPP of the forearm combined with SAL (*n* = 2) or wedge resection (*n* = 9) of the affected arm in a single stage. At a 24-month follow-up, despite no significant circumference reduction being observed at the wrist and hand, a significant circumference decrease was found above (*p* = 0.048) and below the elbow (*p* = 0.02).

For non-compliant patients and those with stage II and III lymphedema, when the stiffened subcutaneous tissue cannot be removed by SAL, the RRPP procedure may prove more effective [129].

#### 7.2.3. Combined Surgical Protocols

Due to the lack of a universally recommended treatment for lymphedema—mainly because of conflicting results reported for each “single-treatment strategy cohort”—surgeons tend to apply surgical techniques based on their personal approach and outcomes. Indeed, a combination of treatment methods could be considered the simplest way to enhance surgical and functional outcomes.

First, manifold clinical phases could co-exist in lymphedema patients, and the occurrence of a dynamic and static condition, namely a lymphatic vessel’s gradual failure and a lymphatic/adipose/fibrotic burden could be recorded. Second, ablative procedures alone are successful in downgrading the lymphatic burden and or the fat/fibrotic collection achieving upper-extremity circumference reduction; though, they are not useful in improving the function of the lymphatic system. Third, the reduction in lymphatic burden, combined with improved lymphatic drainage, may support a synergistic effect. Adding reductive procedures to a physiologic approach helps lower the lymphatic load and limb volume along with the rate of infection, thus improving the quality of life. Moreover, the treatment of lymphedema could be performed simultaneously with breast reconstruction [130].

LVA and VLNT could be associated in a single surgery to achieve a synergistic benefit as they work through alternative mechanisms. LVA offers prompt postoperative improvement through a reduction in the lymphatic load, while VLNT provides long-term lymphedema enhancement by promoting lymphangiogenesis [131].

A combined physiologic treatment method that comprises both VLNT and LVA in a single surgical stage has been proposed by several scholars [131,132,133,134].

In the late stages of BCRL, a combination of both physiological and ablative treatments could be beneficial [135,136,137]. According to the current literature, different timings for employing each technique have been described, and a single-stage approach combining both techniques in one surgical procedure has also been proposed. LVA or VLNT can improve lymphatic drainage while direct excision grants the ablation of pathologic tissue affected by irreversible histologic changes [121,138,139].

The effectiveness of combined VLNT-SAL, and the advantage as a one- or two-stage procedure over VLNT alone have been demonstrated by several scholars. The physiologic procedure helps reduce the lymphatic load of the entire limb, in particular at the most distal part, namely the hand, where ablative procedures cannot be performed [24,139,140]. Moreover, Di Taranto et al. [30] showed that patients with extremity lymphedema treated with an association of VLNT, LVA, and liposuction as a staged protocol sustained a more significant reduction in upper limb circumference compared to those treated only with VLNT and liposuction.

In our recent study, BCRL ISL stage II patients undergoing physiologic procedures (VLNT or LVA) were compared with stage III patients undergoing combined protocols (SAL-LVA or SAL-VLNT). When compared to SAL-LVA (85% ± 10.5%) and to SAL-VLNT (75% ± 8.5%), a more moderate CRR was evident in patients undergoing LVA alone (56.5% ± 8.4%), VLNT alone (54.4% ± 10.2%), or combined VLNT-DIEP flap treatment (56.5% ± 3.9%) even if a reduced disease severity was reported. A comparable rate of complications was evident between groups [99].

Liposuction is not the only reductive procedure that can be safely combined with VLNT for the treatment of BCRL; indeed, the RRPP procedure was safely combined with VLNT showing encouraging results. A single-stage two-team approach consisting of VLNT and concomitant RRPP has been outlined to address late-stage lymphedema, also for patients affected with bilateral extremity lymphedema; in those cases, thorough planning is mandatory [141].

## 8. Nutrition and Lifestyle in the Management of Breast Cancer-Related Lymphedema

The role of nutrition in the prevention and management of BCRL is increasingly recognized as a critical component of a multidisciplinary treatment approach [142]. While surgical and rehabilitative interventions form the cornerstone of lymphedema care, dietary factors may influence the risk, progression, and symptom burden associated with this chronic condition. In particular, nutritional status, dietary patterns, and specific micronutrients can modulate inflammation, immune function, wound healing, and overall patient resilience [143].

Obesity is a well-established independent risk factor for the development and progression of BCRL [144]. Patients with a body mass index (BMI) ≥ 30 kg/m^2^ have significantly higher odds of developing lymphedema compared to those with normal weight. Adipose tissue exerts a dual pathogenic role: it promotes a pro-inflammatory microenvironment and mechanically impairs lymphatic drainage due to fat deposition and interstitial pressure [145]. In addition, obesity exacerbates venous insufficiency and increases the risk of infections, which are common complications in lymphedema patients [146]. Therefore, body weight control should be considered a therapeutic priority in both the prevention and management of BCRL. Dietary counseling aimed at gradual, sustainable weight loss has been shown to improve upper-limb volume, reduce symptom severity, and enhance functional capacity [147,148].

Given the chronic inflammatory nature of lymphedema, anti-inflammatory dietary strategies are of particular interest. The Mediterranean diet—characterized by a high intake of vegetables, fruits, legumes, whole grains, fish, olive oil, and moderate wine consumption—has been associated with reduced systemic inflammation and improved outcomes in cancer survivors [149]. Polyphenols, omega-3 fatty acids, dietary fiber, and monounsaturated fats exert immunomodulatory effects through various mechanisms including modulation of NF-κB signaling and cytokine production [150]. Preliminary evidence suggests that adherence to anti-inflammatory diets may reduce the frequency of cellulitis episodes, improve skin integrity, and ameliorate fatigue and pain. Furthermore, low-glycemic-index diets may help control insulin resistance and adipogenesis, indirectly supporting lymphatic health [151].

Micronutrient adequacy is essential for immune defense and tissue repair in BCRL patients, particularly those undergoing surgical interventions or exposed to recurrent infections [152]. Vitamin D plays a central role in modulating innate and adaptive immunity and has been linked to reduced inflammation and enhanced lymphatic function in experimental models [153]. Likewise, zinc and selenium are critical trace elements with antioxidant and immunoprotective properties that contribute to epithelial integrity and reduce oxidative stress [154]. Although high-quality trials in lymphedema populations are scarce, observational studies suggest that correction of micronutrient deficiencies may improve wound healing, reduce erysipelas recurrence, and promote postoperative recovery [155].

Integrating personalized nutritional counseling into lymphedema rehabilitation programs may provide added value in addressing both physical and psychosocial aspects of BCRL. Registered dietitians should collaborate with oncologists, physiatrists, and surgeons to assess dietary habits, identify nutritional risks, and design tailored interventions. Such counseling can support weight reduction, optimize dietary quality, and promote adherence to anti-inflammatory eating patterns. In addition, nutritional interventions may enhance patient engagement and self-management, which are pivotal for long-term outcomes. While more clinical trials are needed to establish evidence-based guidelines, current recommendations support the incorporation of nutritional strategies as part of a holistic management framework for BCRL. Future studies should explore the impact of specific diets and nutrient supplementation on lymphatic biomarkers, limb volume, and patient-reported outcomes.

## 9. Functioning Assessment Following Treatment for BCRL

Changes in terms of the functioning of BCRL patients undergoing conservative and surgical procedures were evaluated by several studies. The relationship between clinical efficacy—measured as reduced edema or arm volume—and patient outcomes in terms of functional improvement remains somewhat conflicting.

Qiu et al. [156] evaluated 100 patients who underwent LVA and were assessed after a mean follow-up period of 25 months. The mean total lymph ICF score showed a decrease of 13.3 (*p* < 0.001); 43.9 ± 19.0 preoperative to 30.6 ± 20.2 postoperative (*n* = 100) with a decrease in each domain of the lymph ICF. However, a statistically significant decrease (*p* < 0.05) of more than 10 points was observed only in the domains of ‘physical function’ and ‘mental function.’ Arm circumference showed no significant decrease.

Wolfs et al. [157] also evaluated the long-term patency, QoL, and arm circumference in twenty-five patients preoperatively and 12 months after LVA. The lymph ICF questionnaire showed a significant improvement in hand function (*p*  =  0.001), mental function (*p*  =  0.002), and mobility (*p*  =  0.006) 12 months after LVA surgery. A statistically significant improvement was also observed in the total score difference pre- and postoperatively (47.5 and 31.5, respectively; *p* < 0.0001). The household and social domain did not differ significantly. Arm circumference showed no significant decrease.

Cornelissen et al. [106] performed a prospective study aimed at analyzing the effect of LVA on QoL using Lymph-ICF questionnaire. A statistically significant improvement in quality of life, both in the total score and across all QoL domains, was reported after one year of follow-up; however, no significant difference was observed in mean relative volume.

Moreover, Jonis et al. [158] recently performed a prospective randomized multicenter study comparing the effectiveness of LVA versus conservative treatment in women with BCRL. The primary outcome was health-related QoL (HrQoL) measured by the lymph ICF questionnaire (Dutch version) [16]. In the LVA group, the total score of the lymph ICF showed a non-significant difference between baseline and follow-up after three and six months (*p* > 0.05). However, regarding the domains ‘physical function’ and ‘mental function’, a statistically significant improvement was reported. Conversely, in the CDT group, no significant change was observed in the total lymph ICF score between baseline and follow-up. Additionally, arm volume did not show a significant decrease in either group.

Such results should effectively inspire a patient-centered change of paradigm; namely, on the one hand, the focus on functioning could really represent a paradigm shift from surgical outcomes as cold numbers to a patient-friendly evaluation of effectiveness and efficacy of a surgical technique or multidisciplinary protocol. On the other hand, incorporating functional measures into the overall outcome evaluation could provide a more concrete and comprehensive assessment of treatment strategies by taking into account the patient’s subjective experience.

## 10. Limitations

As a whole, the findings of this extensive review highlighted that evidence supporting a specific surgical and rehabilitation treatment are still affected by multiple shortcomings; therefore, intense research is ongoing. On the one hand, the literature dealing with conservative treatment of lymphedema exhibits stronger evidence through several randomized clinical trials [66,67,68]. On the other hand, to date, the surgical treatment of lymphedema has not been investigated through any RCT, but lower-quality studies such as prospective or even case series are often currently proposed. In this scenario, conflicting outcomes and potential biases in the current literature may hinder the development of solid evidence and the establishment of a gold standard.

## 11. Conclusions

According to current clinical practice, the physician’s aims should include improving patient symptoms, minimizing complications, and upgrading functioning. Combined strategies and multidisciplinary protocols for BCRL, including specialized management by plastic reconstructive surgeons and physical and rehabilitative medicine physicians, along with healthy lifestyle programs and personalized nutritional counseling, should be mandatory to address patients’ needs and optimize the treatment of this disabling and non-curable condition. The lymphedema-specific ICF Core Sets should be included more often in the overall outcome evaluation with the aim of obtaining a comprehensive appraisal of the treatment strategies that take into account the patient’s subjective experience.

## Figures and Tables

**Table 1:** ISL staging system for lymphedema [51].

Stage 0	Latent or subclinical condition where swelling is not yet evident despite impaired lymph transport, subtle alterations in tissue fluid/composition, and changes in subjective symptoms. It can be transitory and may exist for months or years before overt edema occurs.
Stage I	An early accumulation of fluid relatively high in protein content (e.g., in comparison with “venous” edema) which subsides with limb elevation. Pitting may occur. An increase in various types of proliferating cells may also be seen.
Stage II	Changes in solid structures are evident. Limb elevation alone rarely reduces tissue swelling, and pitting is manifest. Later in Stage II, the limb may not pit as excess subcutaneous fat and fibrosis develop.
Stage III	Also defined as “lymphostatic elephantiasis”: pitting can be absent and trophic skin changes such as acanthosis, alterations in skin character and thickness, the further deposition of fat and fibrosis, and warty overgrowths have developed.

ISL: International Society of Lymphology.

**Table 2:** Comparison of surgical techniques.

Technique	Aim	Strengths	Drawbacks
LVA	Restore lymphatic circulation Anastomosis L-V	Minimally invasive physiologic procedure Early discharge	Technical challenge Supermicrosurgical expertise and instruments
VLNT	Reestablish natural flow of lymph Transplantation of lymph nodes	Effective also without functional lymphatic vessels Cellulitis reduction Lymphangiogenesis	Risk of donor-site morbidity Longer discharge than LVA
SAL	Fluid and adipose tissue overload removal	Lesser invasive reductive procedure	No function enhancement
RRPP	Adipose tissue and fibrosis removal	More effective than SAL in end-stage lymphedema (fibrosis)	No function enhancement

LVA: lymphatico-venular anastomosis; VLNT: vascularized lymph node transfer; SAL: suction-assisted lipectomy; RRPP: radical reduction with preservation of perforators.

## Data Availability

Data sharing is not applicable to this article as no new data were created or analyzed in this study.
